# Effect of FOLFIRINOX as second-line chemotherapy for metastatic pancreatic cancer after gemcitabine-based chemotherapy failure

**DOI:** 10.1097/MD.0000000000006769

**Published:** 2017-05-12

**Authors:** Noritoshi Kobayashi, Takeshi Shimamura, Motohiko Tokuhisa, Ayumu Goto, Itaru Endo, Yasushi Ichikawa

**Affiliations:** a Graduate School of Medicine, Department of Oncology, Yokohama City University, Japan; b Shimamura Clinic and Yokohama City University Graduate School of Medicine Department of Oncology; c Graduate School of Medicine, Department of Gastroenterological Surgery, Yokohama City University, Japan.

**Keywords:** FOLFIRINOX, gemcitabine, pancreatic cancer, phase I/II study, second-line chemotherapy

## Abstract

**Background:**

This study aimed to determine the maximum tolerated dose (MTD), dose-limiting toxicity, and efficacy of second-line chemotherapy with FOLFIRINOX after gemcitabine (GEM)-based chemotherapy failure in metastatic pancreatic cancer (MPC).

**Methods:**

We studied 18 histopathologically proven MPC patients. The schedule was 85 mg/m^2^ oxaliplatin, irinotecan, and 400 mg/m^2^ leucovorin, followed by 400 mg/m^2^ 5-fluorouracil (5-FU) as a bolus on day 1 and 2400 mg/m^2^ 5-FU as a 46-hour continuous infusion biweekly. The dose of irinotecan was defined as follows: level 0: 100 mg/m^2^, level 1: 125 mg/m^2^, level 2: 150 mg/m^2^, and level 3: 180 mg/m^2^. The doses of other drugs were fixed. The primary endpoint of phase II study was the response rate (RR).

**Results:**

We initially evaluated 6 patients in a phase I study. One patient developed neutropenia and 1 patient developed hyperglycemia and severe infection. Accordingly, level 1 was chosen as the MTD. According to a phase II study, the RR was 22.2% and the disease control rate was 61.1%. The progression-free survival and overall survival were 2.8 (range, 0.7–19.1) and 9.8 (2.4–19.8) months, respectively. The most common severe adverse event was neutropenia (66.7%). Febrile neutropenia occurred in 1 (5.6%) case.

**Conclusion:**

The recommended dose was 85 mg/m^2^ oxaliplatin, 100 mg/m^2^ irinotecan, and 400 mg/m^2^ leucovorin, followed by 400 mg/m^2^ 5-FU as a bolus on day 1 and 2400 mg/m^2^ 5-FU as a 46-hour continuous infusion. These results indicate that second-line FOLFIRINOX is a marginally effective treatment for GEM-based chemotherapy failure cases.

## 1 Introduction

Pancreatic cancer is a relatively frequent malignancy, with a 5-year overall survival (OS) rate of only about 6%.^[1]^ Its incidence has gradually increased over the past 10 years, and >360,000 new cases were projected to occur worldwide in 2015.^[2]^ Metastatic pancreatic cancer (MPC) is one of the most aggressive malignancies. Without treatment, the median survival time is consistently <6 months.^[3]^ Gemcitabine (GEM) has been the standard treatment for metastatic pancreatic cancer since 1997, based on a modest survival benefit compared to bolus 5-fluorouracil (5-FU),^[4]^ and is currently recognized as the standard regimen for MPC.

Various GEM-based combination regimens have also been investigated. Recently, combination treatment of nab-paclitaxel plus GEM improved OS compared with GEM alone in previously untreated patients with MPC (8.5 vs 6.7 months, Hazard Ratio (HR): 0.72, *P* < .001).^[5]^ Hence, GEM-based chemotherapy has remained the standard first-line chemotherapy for MPC worldwide. However, in 2010, a new standard of care emerged when the combination regimen FOLFIRINOX was shown to significantly improve the survival of fit patients with MPC compared with GEM as first-line therapy.^[6]^

A significant percentage (approximately 60%) of MPC patients with relatively good performance status may require second- or even third-line therapy.^[7]^ There are 2 worldwide standard regimens for patients resistant to GEM-based chemotherapy, but there is controversy over their use. The results of a randomized phase III study (the CONKO-003 trial) comparing oxaliplatin/5-FU/folinic acid (OFF) with 5-FU/folinic acid were reported in 2014.^[8]^ OFF was associated with a significantly longer median time to progression (2.9 vs 2.0 months, HR: 0.68, *P* = .019) and median OS (5.9 vs 3.3 months, HR: 0.66, *P* = .010), and is thus considered as second-line treatment for GEM refractory patients in Europe.

Recently, an international phase III study found that nanoliposomal irinotecan with 5-FU and leucovorin extends the survival of patients with MPC who previously received GEM-based chemotherapy.^[9]^ The median OS and progression-free survival (PFS) in patients who received nanoliposomal irinotecan plus 5-FU and leucovorin were 6.1 months and 3.1 months, respectively. According to these 2 randomized phase III studies, 5-FU and leucovorin should be key agents for the second-line treatment of MPC after GEM-based chemotherapy failure.

To our knowledge, only 1 retrospective study has evaluated FOLFIRINOX treatment for MPC patients with disease progression after first-line GEM-based chemotherapy.^[10]^ The aim of the present study was to evaluate the efficacy and safety of second-line FOLFIRINOX treatment in patients with progressive disease following GEM-based chemotherapy, as a prospective phase I/II study.

## 2 Materials and methods

### 2.1 Patients

All patients were aged 18 years or more with histologically or cytologically confirmed metastatic pancreatic adenocarcinoma. Patients who were previously treated with GEM-based first-line chemotherapy were eligible for this study if they met the following inclusion criteria: Eastern Cooperative Oncology Group performance status (PS) of 0 or 1, aged 18 to 75 years, MPC with at least 1 measurable lesion based on the Response Evaluation Criteria in Solid Tumors (RECIST), and adequate hematological, liver, and renal functions (hemoglobin >9.0 g/dL, white blood cell count <10,000/mm^3^, neutrophil count >1,500/mm^3^, platelet count >100,000/mm^3^, total bilirubin <1.5-fold higher than the upper normal limit, serum transaminase <three-fold higher than the upper normal limit, creatinine <1.5-fold higher than the upper normal limit). All patients provided their written informed consent.

Patients were excluded if they had grade 2 or higher peripheral sensory neuropathy, received a blood transfusion, blood products, or hematopoietic growth factor preparations, such as granulocyte-colony stimulating factor (G-CSF) within 7 days before enrolment; had *UGT* genetic polymorphisms (homozygous *UGT1A1∗28* or *UGT1A1∗6* or heterozygous *UGT1A1∗6* and *UGT1A1∗28*); apparent coelomic fluid (pleural effusion, ascites, or pericardial fluid) or peritoneal dissemination; poorly controlled diabetes; synchronous or metachronous double cancer; brain metastases; significant gastrointestinal bleeding or obstruction; or active infection.

This study was initially approved by the Institutional Review Board of Yokohama City University Hospital (B110512020, B130905042) and was conducted according to the Declaration of Helsinki and guidelines on good clinical practice. The clinical trial registration number was UMIN000005808.

### 2.2 Study design

This was an open-label, single-center, nonrandomized, phase I/II study. All laboratory tests required to assess eligibility had to be completed within 7 days prior to the start of treatment.

*Phase I*: The primary endpoint of the phase I study was the determination of the recommended dose for the chemotherapy regimen. The treatment schedule comprised oxaliplatin, irinotecan, and leucovorin on day 1, followed by 5-FU as a bolus on day 1, and 2400 mg/m^2^ 5-FU as a 46-hour continuous infusion biweekly. The doses of oxaliplatin, leucovorin, bolus 5-FU, and continuous 5-FU were fixed (85 mg/m^2^, 400 mg/m^2^, 400 mg/m^2^, and 2400 mg/m^2^, respectively), and the dose of irinotecan was defined as follows: level 0: 100 mg/m^2^, level 1: 125 mg/m^2^, level 2: 150 mg/m^2^, and level 3: 180 mg/m^2^.

Starting at level 1, we planned to test each dose level in 3 to 6 patients. No intrapatient dose escalation was allowed. Dose escalation used a standard “3 + 3” design. The maximum tolerated dose (MTD) was defined as the dose level at which 0 of 3 or 1 of 6 patients experienced dose-limiting toxicity (DLT), with the next highest dose having at least 2 of 3 or 2 of 6 patients encountering DLT during the first 2 cycles. We also evaluated the results of the phase I study as a phase II study.

*Phase II*: The primary endpoint of the phase II study was the response rate (RR), and the secondary endpoints were the OS, PFS, disease control rate (DCR), and safety for all patients, including those involved in the first stage of the study. Pretreatment evaluation using contrast-enhanced computed tomography was performed within 4 weeks before the patient's enrollment. Tumor responses were evaluated every 2 cycles using RECIST version 1.0.^[11]^

### 2.3 Definition of DLTs and dose-reduction criteria of the phase II study

DLTs were determined during the first 2 treatment cycles. DLTs were defined using the Common Terminology Criteria for Adverse Events version 4.0, as one or more of the following effects attributable to the study drug: (1) grade 4 neutropenia lasting longer than 5 days (G-CSF was allowed for grade 4 neutropenia and febrile neutropenia, while pegylated filgrastim was not allowed as primary prophylaxis,) (2) febrile neutropenia, (3) grade 4 thrombocytopenia, (4) any other grade 3 or 4 toxicity, and (5) delay of recovery from treatment-related toxicity for more than 2 weeks. Chemotherapy was delayed until recovery from the following could be achieved: neutrophil count <1500/mm^3^, platelet count <75,000/mm^3^, and total bilirubin >1.5 mg/dL.

The dose-reduction criteria of the phase II study were defined according to the number of adverse events (AEs) following the treatment. At the first, second, and third occurrence of an AE, the bolus 5-FU was removed, the bolus 5-FU was removed and the dose of oxaliplatin was reduced to 60 mg/m^2^, and the study was stopped, respectively. Dose reduction was required for one or more of the following events: (1) grade 4 neutropenia, (2) febrile neutropenia, (3) grade 4 thrombocytopenia, (4) any other grade 3 or 4 toxicity, and (5) delay of recovery from treatment-related toxicity for more than 2 weeks. If there was intestinal pneumonitis of any grade or grade 3 peripheral sensory neuropathy, chemotherapy was stopped. All patients routinely received palonosetron, aprepitant, and dexamethasone for emesis prophylaxis during each cycle.

### 2.4 Statistical analysis

The number of patients to be enrolled was planned using the Southwest Oncology Group (SWOG) standard design (attained design). The null hypothesis was that the overall response rate would be <5% and the alternative hypothesis was that the overall response rate would be >25%; the α error was 5% (one-tailed) and the β error was 20% (one-tailed). The alternative hypothesis was established based on data from previous reports. The sample size was determined as 18 cases. The median survival time and the corresponding 95% confidence intervals (CIs) for OS and PFS were estimated using the Kaplan–Meier method. PFS was defined as the time from day 1 of cycle 1 until the first event (progressive disease or death from any cause). If no such event occurred, data for that patient were censored on the day of the last imaging procedure. OS was defined as the time from day 1 of cycle 1 until death from any cause. In the absence of an event, data were censored on the last day of survival confirmation. All analyses were performed using SPSS version 21.0 (IBM, New York).

## 3 Results

### 3.1 Patients

Between June 2011 and March 2014, 18 patients were enrolled in this study. The patient characteristics at baseline are shown in Table 1. The median age of the patients was 63 (46–68) years. In total, 80% of the patients were classified as having a performance status (PS) of 0, while 20% were classified as having PS 1. Five cases (28%) were pancreas head cancer, 4 of which were treated using metallic stent placement before the study. Eight cases (44.4%) were recurrent tumors after primary resection. The major metastatic sites were the liver and lymph nodes.

**Table 1:**
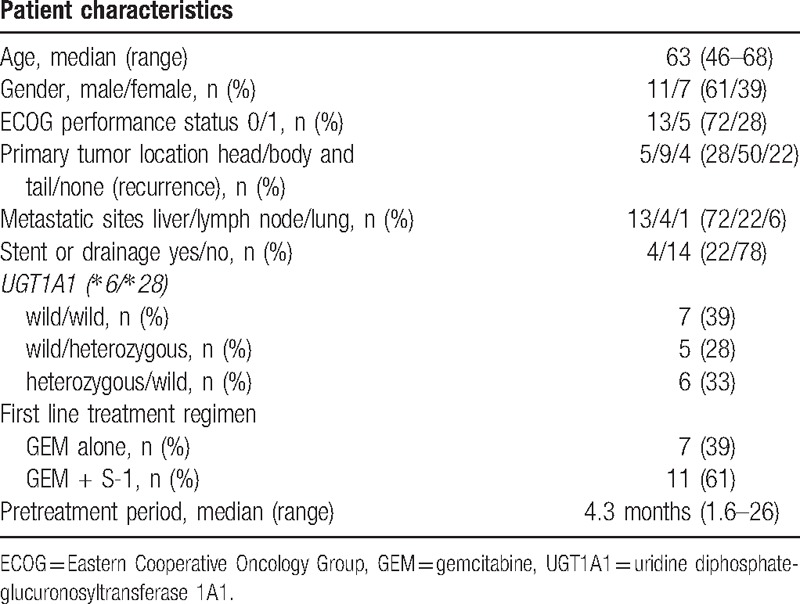
Characteristics of the patients with unresectable pancreatic cancer treated with second-line FOLFIRINOX (n = 18).

All patients received GEM-based chemotherapy (GEM alone: n = 7, GEM plus S-1: n = 11) before enrolling in this study. The median treatment time was 4.3 (1.6–26) months.

### 3.2 Treatment exposure

In level 1 of the phase I study, 8 patients were initially enrolled, and 2 of them were excluded. One patient was excluded because of duodenal perforation that occurred after the first treatment cycle. In this case, the metallic stent in the bile duct injured the duodenal mucosa, and FOLFIRINOX was not related to this severe AE. While this patient recovered by only fasting for 1 week, they were still excluded from the study. The other patient did not develop a severe AE, but chose not to continue the treatment after the first cycle. Among the initially treated 3 patients, 1 patient experienced DLT of grade 4 neutropenia after the first cycle of treatment. In the additionally included 3 patients, 1 patient experienced DLTs of grade 3 severe infection, grade 4 hyperglycemia, and grade 3 thrombocytopenia after the second treatment cycle. This patient had undergone endoscopic metallic stent placement before enrolment into this trial and experienced obstructive cholangitis that necessitated re-endoscopic treatment. The next treatment was performed with a 2-week delay to allow recovery from these toxicities. Accordingly, as 2 of the total of 6 patients showed DLT, we performed dose reduction to level 0 (100 mg/m^2^ irinotecan). In the first 3 cases of level 0, 1 patient experienced DLT of grade 3 anemia, while in the additional 3 cases, there was no DLT. Hence, level 0 was defined as the recommended dose (Table 2). Based on these results, in the phase II study, these 6 patients were enrolled using level 0 irinotecan.

**Table 2:**
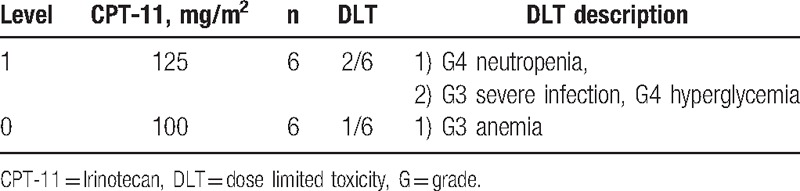
Phase I study of the patients with unresectable pancreatic cancer treated with second-line FOLFIRINOX.

### 3.3 Efficacy

Eighteen patients received FOLFIRINOX, and a complete response, partial response, stable disease, and disease progression were observed in 0, 4, 7, and 7 patients, respectively. The RR was 22.2% and the DCR was 61.1% (Tables 3 and 4).

**Table 3:**
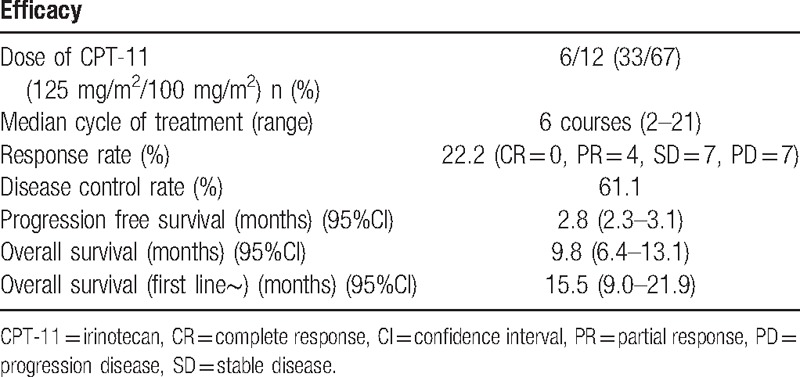
Efficacy results in the patients with unresectable pancreatic cancer treated with second-line FOLFIRINOX (n = 18).

**Table 4:**
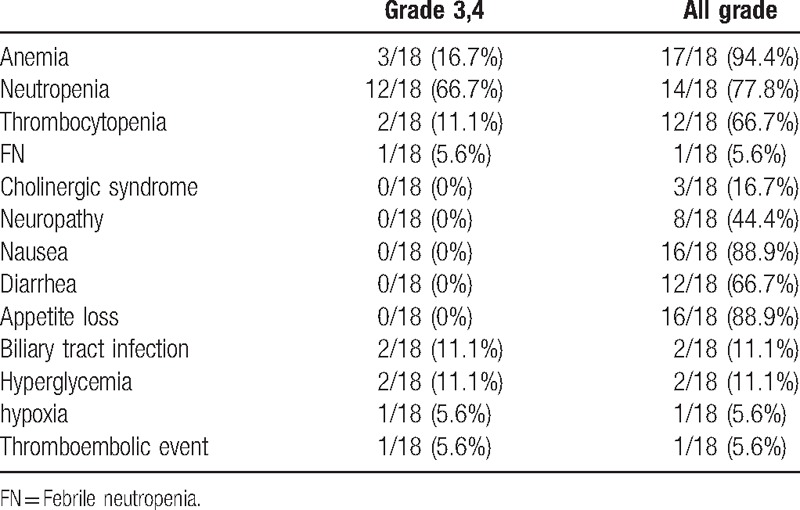
Toxicities in the patients with unresectable pancreatic cancer treated with second-line FOLFIRINOX (n = 18).

**Figure 1:**
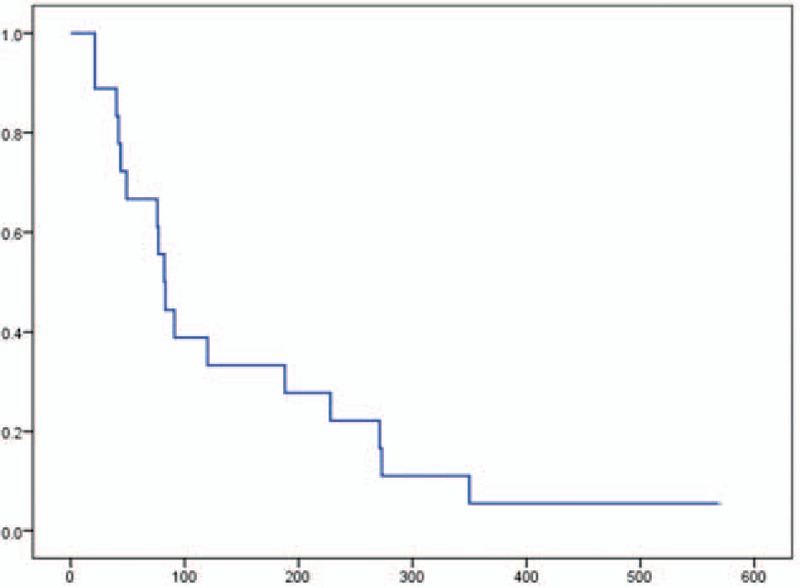
Kaplan–Meier analysis of progression-free survival in a phase II study of FOLFIRINOX as second-line chemotherapy for unresectable pancreatic cancer after gemcitabine-based chemotherapy failure. The median progression-free survival was 2.8 months (95% confidence interval, 2.3–3.1). No patient data were censored.

**Figure 2:**
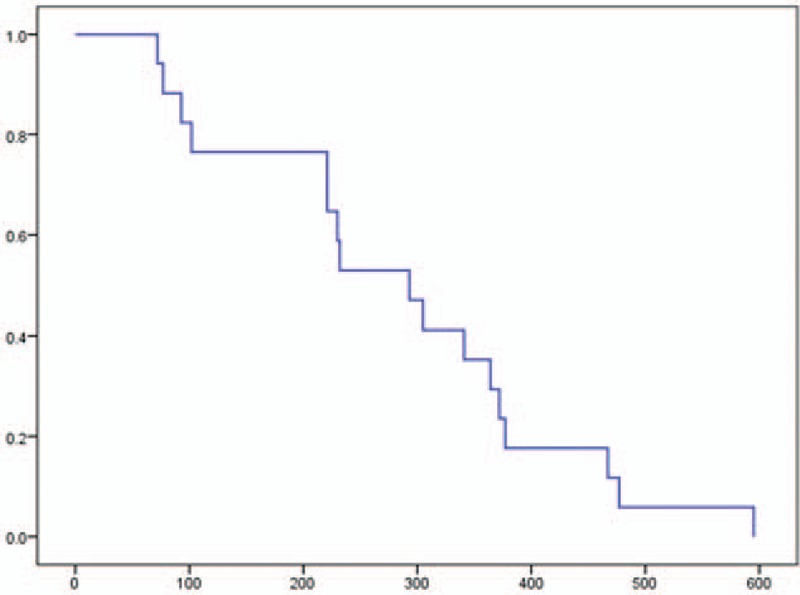
Kaplan–Meier analysis of overall survival in a phase II study of FOLFIRINOX as second-line chemotherapy for unresectable pancreatic cancer after gemcitabine-based chemotherapy failure. The median survival was 9.8 months (95% confidence interval, 6.4–13.1). No patient data were censored.

**Figure 3:**
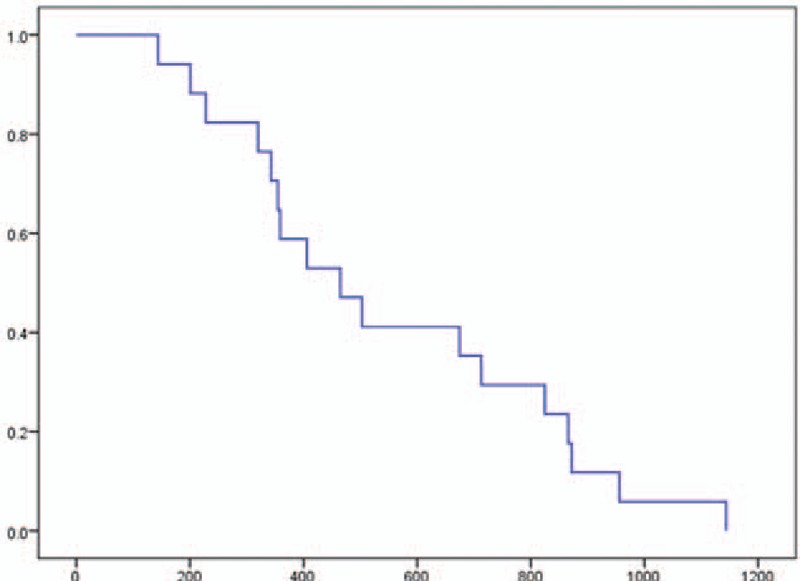
Kaplan–Meier analysis of overall survival from first-line treatment in a phase II study of FOLFIRINOX as second-line chemotherapy for unresectable pancreatic cancer after gemcitabine-based chemotherapy failure. The median survival was 15.5 months (95% confidence interval, 9.0–21.9). No patient data were censored.

The PFS was 2.8 months (95% CI, 2.3–3.1), and the OS was 9.8 months (95% CI, 6.4–13.1). Moreover, the OS from first-line chemotherapy was 15.5 months (95% CI, 9.0–21.9) (Figs. 1–3). At the time of this analysis, all 18 patients had died and none were lost to follow-up.

### 3.4 Safety

There were no treatment-related deaths. Overall, 14 patients (83.3%) experienced grade 3 or 4 AEs (Table 5). The major grade 3 or 4 AEs were hematologic toxicities, such as neutropenia (66.7%). Febrile neutropenia occurred in 2 cases (11.1%). G-CSF treatment was necessary for 7 patients (38.8%). Anemia (16.7%) and thrombocytopenia (11.1%) also occurred. Nonhematological toxicities included appetite loss, nausea, vomiting, and sensory neuropathy, and there were no grade 3 or 4 nonhematological AEs. Cholinergic syndrome related to irinotecan occurred in 3 patients (16.7%). Severe AEs, including bile duct infection (11.1%), hyperglycemia (11.1%), hypoxia (5.6%), and pulmonary artery thrombosis (5.6%), also occurred.

**Table 5:**
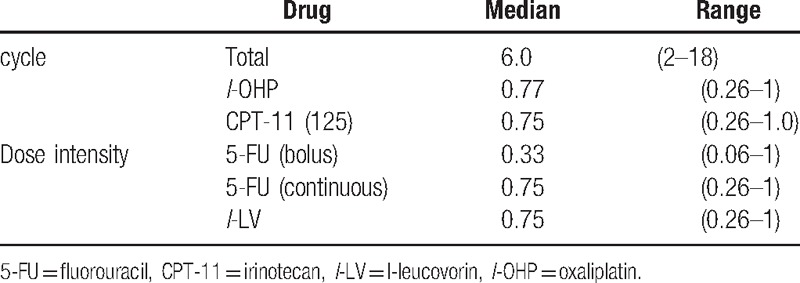
Relative dose intensity of the second-line FOLFIRINOX for unresectable pancreatic cancer.

## 4 Discussion

In the present study, we administered second-line FOLFIRINOX treatment in Japanese MPC patients. We started this study in June 2011; at that time, FOLFIRINOX was not yet approved in Japan for MPC. Moreover, there was no recommended second-line treatment for pancreatic cancer, and full-dose cytotoxic triplet of FOLFIRINOX was considered high risk for Japanese pancreatic cancer patients as the second-line treatment. Furthermore, the AEs of FOLFIRINOX are generally believed to be more severe compared with those of GEM-based chemotherapy, and second-line treatment tends to be associated with more severe AEs than first-line therapy. Accordingly, a feasibility study of FOLFIRINOX for Japanese patients was deemed necessary, and we performed the present phase I study of FOLFIRINOX as second-line treatment for MPC.

Initially, the appropriate dose of irinotecan needed to be established. In the standard regimen of FOLFIRINOX, irinotecan is recommended at a dose of 180 mg/m^2^.^[6]^ However, in Japan, in FOLFIRI treatment for colorectal cancer, the most commonly used dose of irinotecan in clinical practice is 150 mg/m^2^. In a previous study on the recommended regimen of FOLFOXIRI for Japanese metastatic colorectal cancer, the irinotecan dose was 150 mg/m^2^ and bolus 5-FU was omitted.^[12]^ In that study, patients homozygous for *UGT1A1∗28* or *UGT1A1∗6* or heterozygous for both *UGT1A1∗6* and *UGT1A1∗28* were excluded. Based on these previous studies, we determined that level 1 of the irinotecan dose (125 mg/m^2^) of FOLFIRINOX for second-line treatment of pancreatic cancer should be decreased below the commonly used dosage for colorectal cancer. We also excluded patients homozygous for *UGT1A1∗28* or *UGT1A1∗6* or heterozygous for both *UGT1A1∗6* and *UGT1A1∗28*. UGT1A1 is involved in the metabolism of SN-38, an active metabolite of irinotecan, and variants of UGT1A1 have been reported to intensify myelosuppression, such as severe neutropenia.^[13]^

In the present study, at level 1, 2 out of the total of 6 patients showed DLTs, including grade 4 neutropenia, grade 4 hyperglycemia, grade 3 cholangitis (severe infection), and grade 3 thrombocytopenia. In general, obstructive cholangitis and hyperglycemia are not considered direct treatment-related toxicities. In particular, hyperglycemia is considered to be related to the prophylactic use of dexamethasone as an anti-emetic agent. However, in this case, moderate neutropenia and moderate appetite loss continued during treatment; therefore, we decided that these severe conditions were treatment-related toxicities. As a result, in second-line FOLFIRINOX treatment for pancreatic cancer patients, 125 mg/m^2^ irinotecan might be a life-threatening dose. For first-line FOLFIRINOX, a Japanese phase II study reported that the relative dose intensity of irinotecan was 70%, and a study from Yale university similarly reported a relative dose intensity of 64%, that is, doses of approximately 115 to 125 mg/m^2^.^[14,15]^ Herein, we administered this treatment as second-line therapy and did not remove the bolus 5-FU from the regimen; therefore, the recommended dose of irinotecan (100 mg/m^2^) for second-line FOLFIRINOX might be reasonable.

Recently, a number of studies have suggested that modification to the FOLFIRINOX regimen may decrease toxicities without compromising efficacy,^[16–18]^ and, in many studies, the bolus 5-FU was thus removed and/or the irinotecan dose was decreased (165–130 mg/m^2^). Recently, a phase II study of modified FOLFIRINOX for chemotherapy-naïve Japanese patients was reported.^[19]^ This modified FOLFIRINOX regimen comprised 150 mg/m^2^ irinotecan and no bolus 5-FU, and showed improved safety with maintained efficacy without prophylactic pegfilgrastim. In the study by Ueno et al., the relative dose intensity of irinotecan was 89.3% (134 mg/m^2^). In contrast, in our study, we did not plan to exclude the bolus 5-FU, as the dose of irinotecan might have been slightly decreased as a result. Recently, an international phase III study showed that nanoliposomal irinotecan with 5-FU and leucovorin extends the survival of patients with MPC who previously received GEM-based chemotherapy 9). In this study, the dose of nanoliposomal irinotecan was equivalent to 70 mg/m^2^ of irinotecan base every 2 weeks, and the relative dose intensity was 69.8%. Our recommended dose (100 mg/m^2^ irinotecan) might be sufficient as second-line chemotherapy for MPC.

In our phase II study, the primary endpoint (RR) was considered insufficient, and this study became a negative study. However, the RR and DCR were slightly higher than those reported in previous studies (Table 6). FOLFIRINOX is the first triplet regimen investigated in a prospective study on second-line regimens for MPC cases, and we speculate that its cytotoxic effects might contribute to the higher RR and DCR; however, the rate of severe neutropenia might be higher than with other regimens. Moreover, while the PFS did not differ from that of other studies using different regimens, the OS, especially when calculated from the first-line treatment, was longer than previously reported. In our study, the median duration of first-line treatment with GEM or GEM plus S-1 was 4.3 months, which is in accordance with previous studies in which metastatic pancreatic cancer patients received GEM-based chemotherapy for approximately 3 to 5 months.^[8,25]^ Furthermore, in our study, the third-line treatment was not specified, with 9, 4, 1, 2, and 1 cases receiving only best supportive care, GEM monotherapy, nab-paclitaxel plus GEM, GEM plus S-1, and S-1 monotherapy, respectively; however, the median time to treatment failure of these third-line chemotherapies was 1.4 months (data not shown). As we recruited the patients in this study at the time of first-line treatment failure, and because this treatment was not approved in Japan at the time, patients with a relatively good PS might have been selected in this study.

**Table 6:**
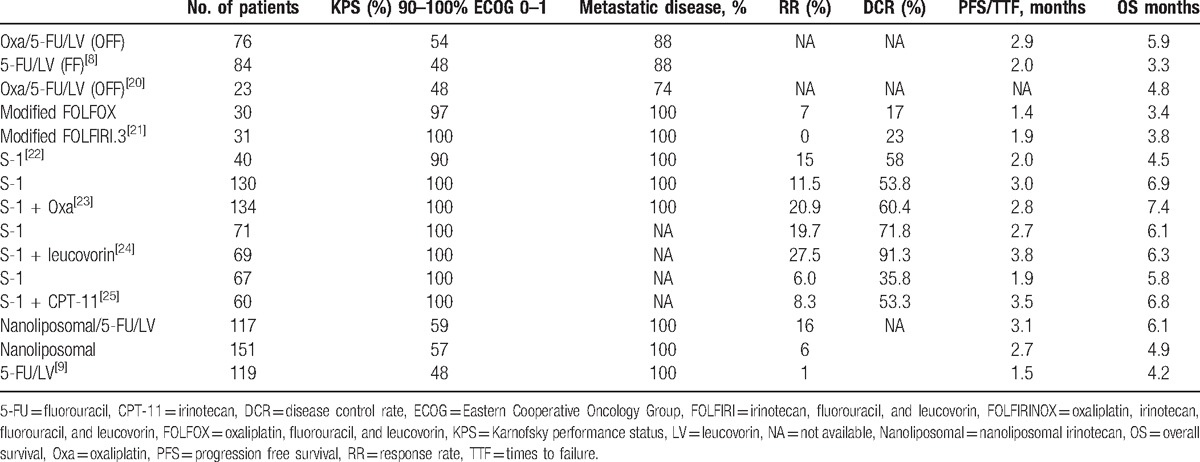
Past reported studies of second-line treatment for unresectable pancreatic cancer.

Both regimens of FOLFIRINOX and nab-paclitaxel plus GEM are effective and feasible treatments for MPC cases. However, there is not enough evidence regarding which regimen to select first. Recently, Portal et al. reported that first-line FOLFIRINOX followed by nab-paclitaxel plus GEM was associated with a median PFS and OS of 5.1 and 8.8 months, respectively;^[26]^ from the start of first-line chemotherapy, the median OS was 18 months. According to these findings, the authors concluded that second-line nab-paclitaxel plus GEM following FOLFIRINOX was feasible and had modest activity and clinical benefit in advanced pancreatic cancer. In our study, there was no case of nab-paclitaxel plus GEM as first-line treatment, with patients receiving either GEM alone or GEM plus S-1. Hence, in the future, prospective studies of second-line FOLFIRINOX following nab-paclitaxel plus GEM are needed.

In conclusion, this is the first prospective study of second-line FOLFIRINOX for MPC worldwide. The results of the present study suggest that FOLFIRINOX is a marginally effective treatment for GEM based chemotherapy failure cases and is feasible as second-line treatment for select advanced pancreatic cancer patients that may be able to prolong OS.

## Acknowledgments

We thank all patients, clinicians, and support staff who participated in this study.
